# Safety and efficacy of different transplant kidney biopsy techniques: comparison of two different coaxial techniques and needle types

**DOI:** 10.1007/s00261-024-04722-w

**Published:** 2024-12-06

**Authors:** Dan Li, Dona Aboud Syriani, Saloni Gupta, James Hui, Jason Hanley, James Sayre, Gary Tse, Frank Hao, Simin Bahrami, Ely Felker, Michael Douek, David Lu, Justin McWilliams, Steven Raman

**Affiliations:** 1 University of California, Los Angeles, USA; 2 Santa Clara Valley Medical Center, San Jose, USA

**Keywords:** Kidney transplant, Interventional radiology, Biopsy complications, Transplant nephrology, Cortical tangential, Core biopsy

## Abstract

**Purpose:**

Percutaneous ultrasound-guided renal biopsy is essential for diagnosing medical renal disorders in transplant kidneys. A variety of techniques have been advocated. The purpose of this study is to evaluate the safety and efficacy of two different coaxial techniques and biopsy devices.

**Methods:**

This single-center dual-arm, observation study cohort included 1831 consecutive transplant kidney biopsies performed over a 68-month period. Two coaxial techniques were used, distinguished by whether the 17 gauge (G) coaxial needle was advanced into the renal cortex (intracapsular technique; IC) or to the edge of the cortex (extracapsular technique; EC). One of two needle types could be used with either technique: an 18G side-cutting (Bard Max-Core or Mission) or an 18G end-cutting (Biopince Ultra) needle. In all cases, the cortical tangential technique was used to reduce the risk of central artery transgression and unnecessary medullary sampling. Patients were monitored for 30 days post-procedurally and complications were evaluated using the SIR adverse event classification.

**Results:**

Of the 1831 patients included in the study cohort, 13 suffered severe bleeding complications requiring operative intervention. Of these patients, 8 underwent biopsy with side-cutting needle and IC, 2 with side-cutting needle and approach not specified, 2 with end-cutting needle and IC, and 1 with end-cutting needle and EC. There was no statistically significant difference in the risk of bleeding complications between different coaxial techniques and needle types. However, there was a significantly increased chance of inadequate sampling when comparing the side-cutting needle (1.0%) to the end-cutting needle (0.1%).

**Conclusions:**

Transplant kidney biopsy performed with two different coaxial techniques and needle types did not show differences in bleeding complications. There is an increased risk of inadequate sampling when using side-cutting relative to end-cutting biopsy devices.

## Introduction

Percutaneous image guided transplant renal biopsy is essential for providing valuable diagnostic and prognostic information in transplant patients. A variety of techniques have been described for performing percutaneous renal biopsies since the early 20th century, including IV pyelography guided percutaneous needle biopsy in 1951, first developed to supplant earlier palpation guided biopsies [1, 2]. Techniques have evolved and now are typically guided by ultrasonography and computed tomography (CT) with biopsy needles advancing from cutting needles to spring-loaded automatic or semi-automatic needles [3, 4].

**Fig. 1:**
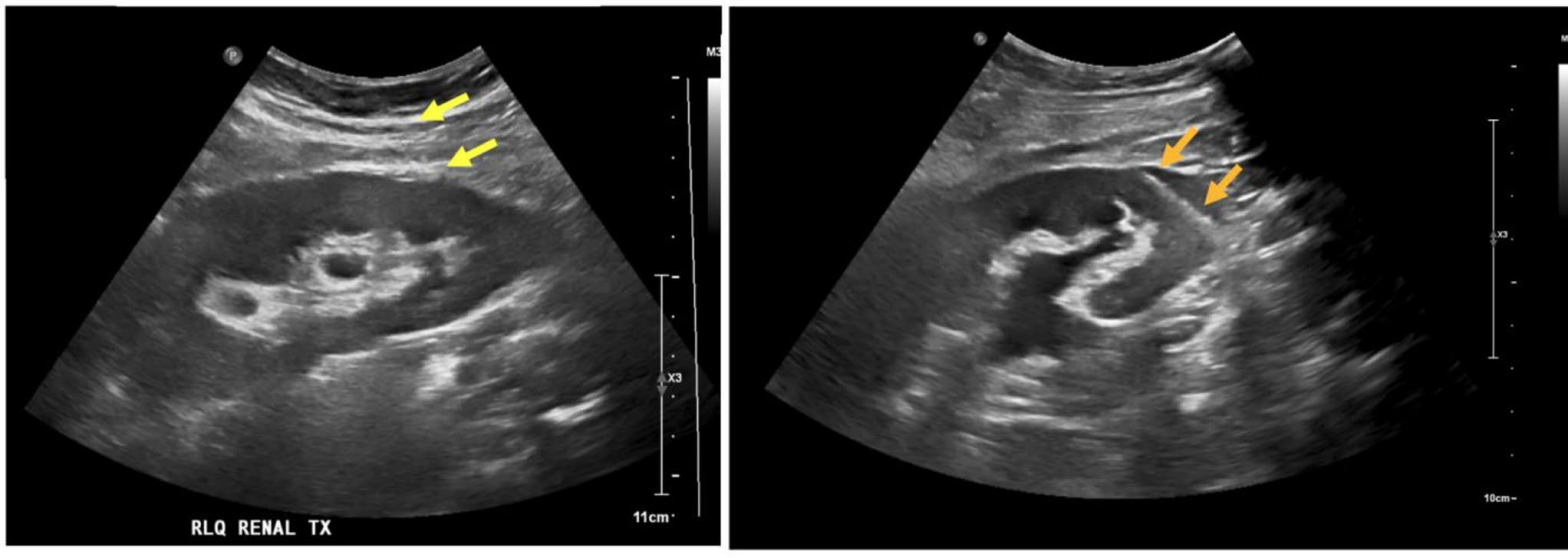
Cortical tangential approach. Yellow arrows: 17G co-axial introducer parked outside the transplant kidney lower pole (Extra-capsular Technique). Orange arrows: 18G Biopince Ultra end-cutting device fired through the lower pole of the kidney, away from the collecting system and medulla. A similar cortical tangential approach would be used with the side-cutting needle, though the co-axial may be advanced just into the renal cortex in these cases

Percutaneous needle biopsy of renal transplant for diagnosis of rejection, acute tubular necrosis or other nephropathies requires obtaining a minimum number of intact glomeruli for adequate histopathologic and electron microscopy evaluation [5, 6]. PNB must balance tissue acquisition against risk of bleeding complications which are associated with low platelet count, elevated prothrombin time, number of biopsy needle passes, biopsy needle size, female gender, and elevated creatinine [7–9]. Changes in needle trajectory including the “cortical tangential approach” to renal biopsy have been advocated to maximize tissue yield and minimize bleeding complications [10, 11]. The cortical tangential approach describes the technique of paralleling the upper or lower pole of the cortex, while remaining in the outer third to half of the distance between the cortex and renal hilar fat, avoiding excess medullary sampling and potential injury to hilar structures (Fig. 1). Excess renal medullary sampling should be avoided because it is unhelpful for diagnosis (per the Banff 97 pathologic criteria) but may necessitate additional core samples which increase the risk of hemorrhage and arterio-venous fistula formation [6]. Additional discussion has arisen debating the relative safety of various coaxial introducer techniques, resulting in the development of both intracapsular and extracapsular approaches. During the intracapsular approach, the coaxial introducer needle is advanced through the renal capsule into the renal cortex; in contrast, during extracapsular approach, the proceduralist leaves the coaxial needle just outside the renal cortex with only the spring-loaded biopsy needle traversing across the renal capsule. Different types of biopsy devices - broadly divided into side cutting and end cutting needles - have been recommended to maximize tissue yield while minimizing number of biopsies (Fig. 1). Both types of coaxial approach and biopsy devices are used by abdominal and interventional radiologists at this institution, presenting a unique opportunity to compare their safety and efficacy. The purpose of this study is to assess the risk of bleeding complications and sampling efficacy of two different needles and coaxial techniques used in renal transplant biopsy.

## Methods/materials

### Patient population

This single-center, IRB-approved retrospective observational study included all adult patients (age ≥ 18) who underwent ultrasound guided transplant kidney biopsy over a 68-month period (January 3, 2017 through September 16, 2022), which totaled 1831 patients (1148 men, 681 women; age range 18–83 years; mean age, 50 years). The study was compliant with the Health Insurance Portability and Accountability Act. Informed consent was not required per institutional IRB and policies for this retrospective analysis.

### Procedural details

Prior to procedure, serum laboratory values including complete blood count (CBC), prothrombin time (PT), partial thromboplastin time (PTT), international normalized ratio (INR) and basic metabolic panel (BMP) were routinely obtained. Patients with abnormal coagulation parameters such as elevated PT were corrected to 1.5 times normal with intravenous Vitamin K. Those with elevated PTT were corrected with fresh frozen plasma (FFP). Those with platelets less than 50 × 10^9^/L received a platelet transfusion in accordance with Society of International Radiology (SIR) procedural guidelines [12]. Patients on anticoagulants withheld their medications according to the SIR management recommendations for high-risk bleeding procedures (i.e. Coumadin 5 days prior, aspirin 3–5 days prior) and were advised to re-start anticoagulation 12–48 h following biopsy (patient dependent) [12]. Patients with elevated serum blood urea nitrogen (BUN) > 20 were given intravenous DDAVP. Pre-procedure blood pressure goals were set at a systolic pressure less than 160 mm Hg and diastolic pressure below 90 mm Hg. For acute elevations in blood pressure, intravenous antihypertensive medications were administered. Biopsies were occasionally performed at higher pressures in patients with chronic blood pressure elevations per operator preference.

Most procedures were performed by a staff abdominal or interventional radiologist supervising a radiology fellow or resident in a dedicated procedure suite, with a minority of biopsies performed by the staff radiologist independently. In the supine position, all patients underwent either a full or abridged ultrasound scan of the transplant kidney with grayscale, color, and power Doppler evaluation of the renal parenchyma. Based on the ultrasound and additional scanning by the physicians, a suitable site was chosen for access to the upper or lower pole of the transplant kidney and the area cleansed with 1% chlorhexidine and draped with sterile blue towels and full drape of the lower extremities. For local anesthesia, 1% lidocaine was injected with a 25G needle subcutaneously and to the transplant renal capsule.

**Fig. 2:**
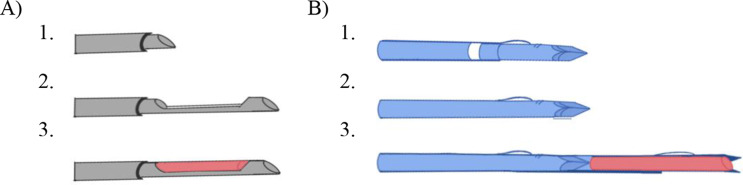
Side-cutting versus End-cutting needle. Schematic drawings illustrating the mechanism of two different core biopsy devices. (**A**) Traditional side-cutting biopsy device captures a semi-cylindrical core of tissue (i.e. Bard Mission and Bard Max-Core, CR Bard). (**B**) End-fire biopsy device captures a complete cylinder of tissue (i.e. Biopince, Argon medical)

With ultrasound guidance, a 17G coaxial introducer needle was advanced to the transplant renal capsule. Depending on the attending radiologist preference, either an extracapsular or an intracapsular technique were used with either an 18G side-cutting biopsy needle (Mission or Max-Core, Bard, Murray Hill, NJ) or an 18G end-fire biopsy needle (Ultra, Biopince, Argon Medical, Plano, TX) was used (Fig. 2). The extracapsular technique consisted of guiding the 17G coaxial introducer needle to the renal transplant capsule with ultrasound guidance, followed by 1–2 punctures of the outer renal cortex with 18G biopsy device (typically Biopince Ultra). The intracapsular technique consisted of guiding the 17G needle approximately 5 mm into the renal cortex with ultrasound guidance, followed by 1–2 punctures of the outer renal cortex with 18G coaxial biopsy device (typically Bard Mission or Max-Core). This resulted in four total possible techniques: Intracapsular coaxial technique/side-cutting needle, intracapsular coaxial needle/end-cutting needle, extra-capsular coaxial technique/side-cutting needle, and extra-capsular coaxial technique/end-cutting needle. Regardless of which technique or biopsy needle was used, one tissue sample was obtained and analyzed by the on-site pathology technologist who evaluated the number and quality of glomeruli under light microscopy for diagnostic adequacy and determined if a second core was indicated. In most cases, two samples were obtained. Following biopsy, at the discretion of the performing attending, 1–3 gel-foam pledgets were loaded into a 1 cc syringe and injected through the 17G outer cannula. Pressure was applied above the transplant kidney for hemostasis. Finally, participants were monitored with ultrasound for up to 5 min to evaluate for immediate complications. They were then taken or back to a post-procedural area (outpatients) or returned to inpatient bed and instructed to remain supine for 3 h. No routine delayed renal ultrasound, urinalysis, or blood tests were performed unless there was symptomatic change or other clinical indication.

### Statistical analysis

Pre-procedure serum laboratory values, biopsy technique, biopsy device, number of cores, and sufficiency of biopsy sample were recorded. Patients were followed for bleeding complications up to one month after the procedure using SIR adverse event classification [13]. Major complications included patients requiring invasive intervention, either minor or major hospitalization, or permanent adverse sequelae. Minor complications included incidental findings attributed to the biopsy without clinical consequence, as well as patients requiring nominal therapy or overnight admission for monitoring.

Statistical analysis was performed using SPSS 28.0.1 for Windows (IBM, Armonk, New York). Fisher’s exact tests were performed to compare complication rates and biopsy sample sufficiency between the four sub-cohorts (extra-capsular technique with end-cutting needle, extra-capsular technique with side-cutting needle, intra-capsular technique with end-cutting needle, and intra-capsular technique with side-cutting needle). Additional Fischer’s exact tests were performed to compare complication rates and biopsy sample sufficiency between intra-capsular versus extra-capsular technique and the side-cutting versus end-cutting needle sub cohorts. Finally, comparison was made between patients with bleeding complications and patients without using a combination of independent t-test (Age, INR, Platelets) and Fischer’s exact test (whether tract embolization was performed). Two-tailed p-values less than 0.05 were considered statistically significant.

## Results

Of the 1831 participants meeting the above criteria, 1039 biopsies were performed were performed using a side-cutting needle and 785 samples were performed using an end-cutting needle. The co-axial approach was specified in 1364 of these cases (intracapsular versus extracapsular). Out of these patients, 912 participants underwent intracapsular puncture and 452 participants were biopsied using the extracapsular technique. The majority of the end-cutting device samples were obtained using the extracapsular technique (404 out of 452) and nearly all the side-cutting device samples were obtained using the intracapsular technique (779 out of 912). No significant difference between groups defined by coaxial technique or needle type was found for age, INR, or platelet count (Table 1). There was a significant increase in the number of cores obtained using the side-cutting needle when compared to the end-cutting needle (2.21 vs. 2.09, *p* < 0.01).

**Table 1:** Patient characteristics

	**Intra-capsular technique** **(*n*** = **912)**	**Extra-capsular technique** **(*n*** = **452)**	***P*** **-Value**
Age	50.2	50.5	0.73
INR	1.07	1.08	0.31
Platelets	205.7	207.6	0.66
Cores	2.21	2.09	< 0.01
	**Side-cutting needle** **“Bard”** **(*n*** = **1039)**	**End-cutting needle “Biopince”** **(*n*** = **785)**	***P*** **-Value**
Age	50.0	50.8	0.25
INR	1.07	1.08	0.18
Platelets	207.3	205.6	0.62
Cores	2.20	2.19	0.87

Two-sided t-tests were used to calculate differences between age, INR, platelets, and number of cores taken between biopsies performed using different coaxial techniques and biopsy devices

Severe bleeding complications occurred in 13 participants. Of these patients, 1 patient was lost to follow-up and 4 experienced sequelae of renal injury which contributed to graft failure. There was no difference in the age, pre-procedural laboratory values, or number of cores taken in participants who suffered severe bleeding complications versus those who did not (Table 2). Out of the 13 patients who suffered severe bleeding, 8 underwent biopsy with a side-cutting needle and intra-capsular puncture, 2 underwent biopsy with a side-cutting needle with intra versus extra-capsular approach not specified, 2 underwent biopsy with end-cutting needle with intra-capsular puncture, and 1 underwent biopsy with end-cutting needle and extra-capsular approach.

**Table 2:** Patient characteristics between patients with and without complication

	Severe bleeding complication (*n* = 13)	No severe bleeding (*n* = 1818)	*P*-value
Age	48.9	50.4	0.72
INR	1.14	1.08	0.04
Platelets	207	181	0.10

Two-sided t-tests used for age. One-sided t-tests used for INR and platelets

There was no significant difference in mild, moderate, or severe bleeding complications between the four sub cohorts (Table 3). Additional two-way sub-cohort analysis comparing only the two needle types and only the two coaxial types showed an increase in the risk of inadequate sampling when using the side-cutting needle (1.0% vs. 0.1%, *p* = 0.03; Table 4). There was a significant increase in mild bleeding complications when using either the end-cutting needle versus the side-cutting needle (1.8 vs. 0.6%, *p* = 0.02; Table 4), though no difference in moderate or severe bleeding complications. Of the 13 patients that suffered severe bleeding complications, 12 received gelfoam tract embolization; however, correlation between gelfoam use and severe bleeding did not reach statistical significance when compared using Fischer’s exact test (*p* = 0.09).

**Table 3:** Complication rate and sample adequacy between four unique techniques

	Extra-capsular/end-cutting (*n* = 404)	Intra-capsular/end-cutting (*n* = 125)	Extra-capsular/side-cutting (*n* = 48)	Intra-capsular/side-cutting (*n* = 779)	*P*-value
Mild Complication (Immediate post-procedural hematoma)	1.7%	0%	2.1%	0.6%	0.15
Moderate complication (Blood transfusion within 30 days)	5.3%	7.8%	13.0%	7.8%	0.59
Severe complication (OR intervention)	0.2%	0.8%	0%	0.9%	0.56
Inadequacy (Proportion of samples deemed inadequate by pathology)	0.2%	0%	0%	1.1%	0.22

Fischer’s exact test was used to evaluate for differences between the four sub-cohorts (End-cutting/Extra-capsular, End-cutting/Intra-capsular, Side-cutting/Extra-capsular, Side cutting/Intra-capsular). Biopsies where the coaxial technique was not specifically described in the operative report are excluded

**Table 4:** Complication rate and sample adequacy between biopsy needle types and between extracapsular and intracapsular technique

	**End-cutting needle** **(*n*** = **1037)**	**Side-cutting needle** **(*n*** = **785)**	***P*** **-value**
Mild Complication (Immediate post-procedural hematoma)	1.8%	0.6%	0.02
Moderate complication (Blood transfusion within 30 days)	7.3%	8.5%	0.43
Severe complication (OR intervention)	0.4%	1.0%	0.17
Inadequacy (Proportion of samples deemed inadequate by pathology)	0.1%	1.0%	0.03
	**Extra-capsular technique** **(*n*** = **452)**	**Intra-capsular technique** **(*n*** = **912)**	***P*** **-value**
Mild Complication (Immediate post-procedural hematoma)	1.8%	0.6%	0.04
Moderate complication (Blood transfusion within 30 days)	5.5%	7.8%	0.19
Severe complication (OR intervention)	0.2%	0.9%	0.29
Adequacy (Proportion of samples deemed inadequate by pathology)	0.2%	1%	0.18

Fischer’s exact test was used to compare end-cutting and side-cutting sub-cohorts, and then separately to compare extra-capsular coaxial technique versus intra-capsular coaxial technique sub-cohorts

## Discussion

Percutaneous ultrasound guided transplant kidney biopsy has been shown to be a safe, convenient, and cost-effective technique. At least one prior study has demonstrated a high diagnostic yield utilizing an end-cutting needle taking only a single core sample, compared to the higher number of cores traditionally obtained utilizing side-cutting needles [10]. For this reason, it is thought that the end cutting needle may also be superior for bleeding complications. At this institution, use of the extra-capsular coaxial technique is theorized to reduce the risk of bleeding complication by decreasing the diameter of the capsule puncture (i.e. 18G puncture from the biopsy needle only when using an extra-capsular technique versus 17G puncture from the co-axial device when using an intra-capsular technique).

This study demonstrated that proceduralists using the end-cutting needle obtained a significantly lower number of cores on average (2.09 versus 2.21), although this difference did not persist when comparing co-axial techniques. This may suggest that the end-cutting cores were more likely to have a higher number of glomeruli, as the samples were verified prior to completing the procedure.

Both techniques delivered a very high diagnostic yield, with only 12 samples out of 1831 later found to be inadequate by the pathologist after delivery to the laboratory. Only 1 of the inadequate samples (0.1%) was obtained using the end-cutting needle, while the remainder (1.0%) were obtained using a side-cutting device. Despite taking a lesser number of cores on average, the end-cutting needle was more likely to yield adequate tissue sampling. These findings reinforce Patel et al’s hypothesis that end-cutting needles deliver higher diagnostic yield using an equivalent or lesser number of cores [11].

The risk of post-procedural bleeding has been described in the literature ranges from 0.1 to 7.4%, which was congruent with his dataset, where 0.7% of participants experienced severe bleeding complications. There was no significant difference in moderate or severe bleeding complications between different techniques. Interestingly, there was increased risk of mild bleeding complications (immediate post-procedural hematoma) in the two-way sub-cohort analysis when comparing the end-cutting needle to the side-cutting needle. Of the 20 immediate post-procedural bleeding complications recorded, 7/20 occurred under the same radiologist (the remaining 13 complications occurred between 6 other proceduralists). This radiologist did not have a disproportionate number of moderate or severe complications and none of these immediate hemorrhages required operative intervention. In this case, the increased risk of immediate complications may have been due to lower threshold to report very small hematomas and/or greater than average post-procedural monitoring period. There was no correlation between tract embolization with gelfoam and risk of severe bleeding complications.

The power of this study was limited by the low number of adverse events in all sub-cohorts, which may have prevented the number of severe bleeding complications from reaching statistical significance. Direct comparison between needle types and coaxial techniques (as performed in Table 4) may also suffer from cofounding effects. For instance, certain providers may favor specific combinations of coaxial techniques and needle types. Similarly, gelfoam was generally deployed by radiologists using intracapsular coaxial technique, limiting conclusions about the benefit of tract embolization. Utilization of a pathology technician or pathologist to review the samples prior to completing the procedure allowed clinicians to overall obtain a lower number of cores than has been described with previous studies, particularly with the side-cutting needle, which may have further obscured small differences in safety and sample efficacy between the two techniques. Continued evaluation of safety outcomes with even a larger cohort may help elucidate these small, but possibly significant, differences.

Use of a coaxial needle in all renal biopsies is utilized at this institution because it allows additional samples to be obtained without having to re-target the renal cortex following real-time verification by on-site pathologist/pathology technician. However, it is worth noting that other institutions perform these biopsies forgoing the co-axial introducer. Additionally, all biopsies at this institution are performed with an 18G biopsy device, limiting comparisons of safety and adequacy between different diameter devices.

The primary endpoint of this review evaluated sample adequacy in a binary way. However, subjective preferences within the pathology department for tissue samples obtained with different devices may exist. This reveals a possible avenue for future research, which could further inform proceduralists’ preferences for specific biopsy devices.

## Conclusion

Performing transplant kidney biopsy with an end-cutting needle was more likely to yield adequate tissue sampling when compared to a side-cutting needle, though both devices demonstrated high adequacy rates when real time tissue yield verification was performed. There were no significant differences in the rate of severe bleeding complications using any combination of intra- and extra-capsular coaxial techniques with either needle type.

## Data Availability

No datasets were generated or analysed during the current study.
